# GAHLS: an optimized graph analytics based high level synthesis framework

**DOI:** 10.1038/s41598-023-48981-x

**Published:** 2023-12-19

**Authors:** Yao Xiao, Shahin Nazarian, Paul Bogdan

**Affiliations:** University of Southern California, Los Angeles, CA 90089 USA

**Keywords:** Electrical and electronic engineering, Computer science

## Abstract

The urgent need for low latency, high-compute and low power on-board intelligence in autonomous systems, cyber-physical systems, robotics, edge computing, evolvable computing, and complex data science calls for determining the optimal amount and type of specialized hardware together with reconfigurability capabilities. With these goals in mind, we propose a novel comprehensive graph analytics based high level synthesis (GAHLS) framework that efficiently analyzes complex high level programs through a combined compiler-based approach and graph theoretic optimization and synthesizes them into message passing domain-specific accelerators. This GAHLS framework first constructs a compiler-assisted dependency graph (CaDG) from low level virtual machine (LLVM) intermediate representation (IR) of high level programs and converts it into a hardware friendly description representation. Next, the GAHLS framework performs a memory design space exploration while account for the identified computational properties from the CaDG and optimizing the system performance for higher bandwidth. The GAHLS framework also performs a robust optimization to identify the CaDG subgraphs with similar computational structures and aggregate them into intelligent processing clusters in order to optimize the usage of underlying hardware resources. Finally, the GAHLS framework synthesizes this compressed specialized CaDG into processing elements while optimizing the system performance and area metrics. Evaluations of the GAHLS framework on several real-life applications (e.g., deep learning, brain machine interfaces) demonstrate that it provides 14.27× performance improvements compared to state-of-the-art approaches such as LegUp 6.2.

## Introduction

The end of Moore’s law motivates hardware designers to pack heterogeneous processing elements (PEs) (e.g., general-purpose processors (GPPs), graphics processor units (GPUs), digital signal processors (DSPs), application-specific hardware accelerators (ASHAs), coarse-grained reconfigurable architectures (CGRAs)) on single chips, namely domain-specific systems on chip (DSSoC), to allow higher system scaling while improving the system performance and limiting the maximum energy consumption. Therefore, to match the requirements of complex emerging applications in autonomous systems, cyber-physical systems, Internet-of-Things, advanced robotic systems capable of creative problem solving and aiming to ensure a higher degree of design productivity, the complexity of hardware/software co-design and chip (hardware) design is rapidly changing to boost performance and energy efficiency. At the same time, due to the era of big data and complex data science (e.g., multimodal sensing and Internet-of-Things (IoT), wearables, machine learning, artificial intelligence, federated learning, autonomous systems, evolvable computing), software is rapidly evolving to allow for programming complicated perception, inference, analysis, and decision-making tasks. For example, remarkable research progress has been made in the robotics field from deep-see exploration to outer space^1^. However, most of these research efforts in the areas of cyber-physical systems, autonomous systems, cyber-human-physical systems and advanced robotic systems rely on the advancement of software algorithms such as exploration, navigation and planning in dynamic environments^2^ and brain-machine interfaces^3–5^. One of the major challenges remained in robotics and insect-inspired AI^6^ is still energy expenditure and on-board intelligent processing speed^1^, meaning that the robots should be able to accomplish the complex collective computational tasks as soon as possible within a limited power budget, especially for the embedded and mobile robots. Different applications require different types of computing hardware, with processing speed including the parallel architectures such as the GPU and FPGA. Therefore, one of the contributions in this work is to intelligently synthesize parallel (domain-specific) accelerators (DSAs) for higher performance and energy efficiency.

**Figure 1:**
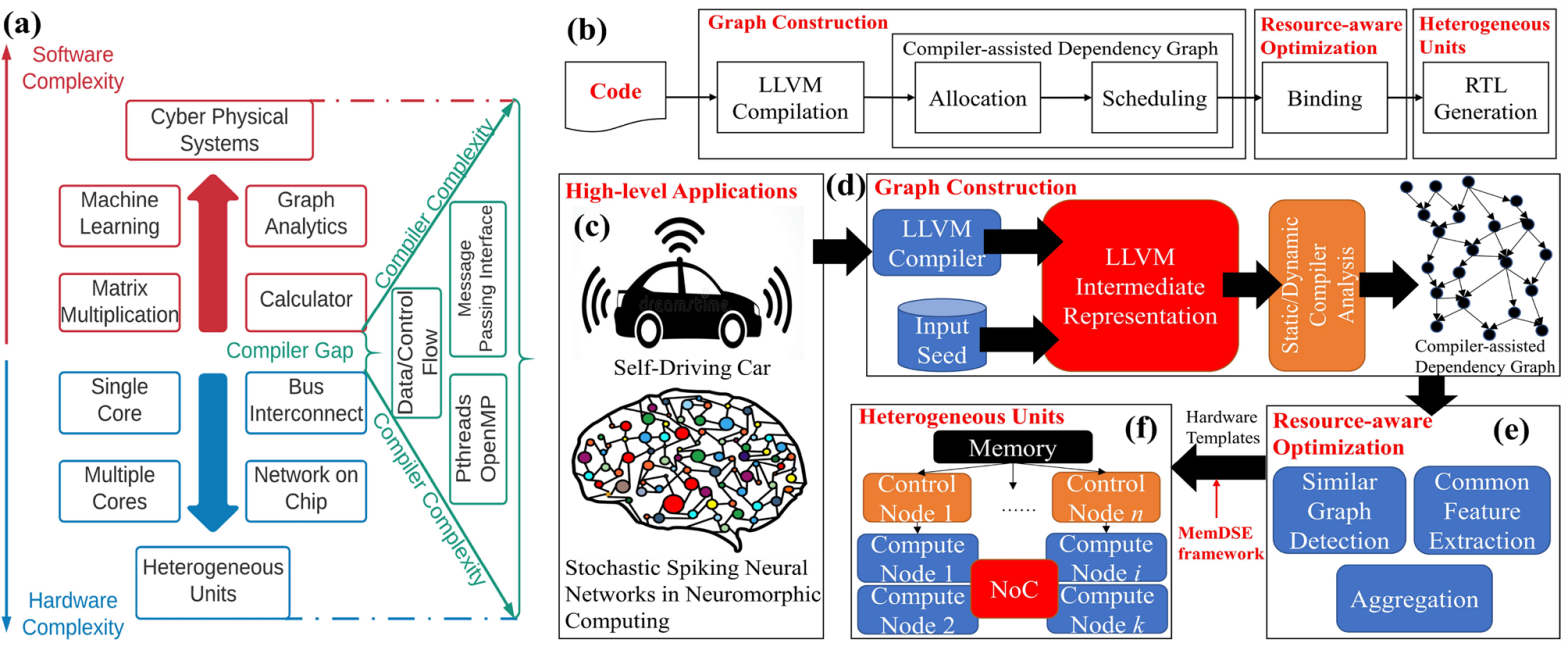
Autonomous graph analytics-based high level synthesis framework for promoting parallelism in heterogeneous on-board intelligent AI systems. (**a**) Software and hardware complexity gap. Due to multimodal rich sensing and big data, the software is rapidly growing in functional complexity and computational requirements enabling numerous applications in diverse areas such as medical and autonomous systems. At the same time, the end of Moore’s law contributes to higher hardware complexity consisting of heterogeneous hardware units (implemented in diverse technologies). This increase in complexity makes the compiler gap severe for complex high-level programs to be efficiently executed on hardware. (**b**) The typical HLS workflow with emphasis on our synthesis framework (**c**–**f**) that automatically translates high-level programs (**c**) into agile hardware-level source code description. It consists of the following steps: (**d**) We collect dynamic LLVM execution traces from a given application to be accelerated on hardware and construct a compiler-assisted dependency graph (CaDG) via static and dynamic data and control dependencies. (**e**) We perform a three-stage resource-aware optimization consisting of similar subgraph detection, common feature extraction, and aggregation, to find similar subgraphs which can be merged to reduce the resource usage. (**f**) Following the control and compute node templates, we convert the optimized CaDG-based functional representation into heterogeneous units, where nodes represent either computation or function calls (control nodes).

Synchronization overhead and workload imbalance in parallel programming on multiple heterogeneous cores can exacerbate the system performance written in programming interfaces such as OpenMP^7^ and message passing interface (MPI)^8^. The compiler fails to compile the efficient machine code for such applications^9^. As shown in Fig. 1a, the compiler gap, defined as the difference between software and hardware complexity, is increasing and making it extremely difficult for the compiler to decide for a given software program which portions to be mapped on GPUs and the rest to CPUs. Software programmers have to explicitly insert *pragmas* to ask for compiling a piece of code into GPU. In order to unify the software and hardware, we propose a novel *compiler framework* to design heterogeneous units for a given application, without requiring human reasoning analysis, intervention, and annotation.

Moreover, the hardware accelerators are usually more energy and time efficient compared to running software on the traditional CPUs/GPGPUs. This is because the accelerators are tailored to one algorithm whereas CPUs/GPGPUs are general-purpose machines, capable of running a wide range of applications. It is beneficial to design heterogeneous accelerators on-a-chip where CPUs offload specialized tasks onto accelerators to improve performance^10–12^. Therefore, in order to reduce the programmer’s burdens, several tools such as Vivado high-level synthesis (HLS) and LegUp^13^ synthesize high-level languages (HLLs) (e.g., C, C++) into hardware design languages (HDLs) without prior knowledge on hardware design. However, this effort faces the following challenges: First, most existing HLS tools still require programmer knowledge to insert proper *pragmas* into HLLs such as “#pragma HLS unroll” in Vivado HLS, which makes these tools complicated to use. Second, although some tools can generate HDL without pragmas, the synthesized hardware is not optimized to discover the optimal parallelization and take full advantage of the intrinsic concurrency of a complex software of an AI system in the context of available heterogeneous hardware resources.

The overarching **goal** of this paper is to identify heterogeneous tasks hidden in the complexity of high level programs that can benefit from running on heterogeneous processing units and propose a resource-aware optimization to select the best target processing units for these tasks. We build a novel compiler framework for generating optimized hardware accelerators from HLLs without pragmas (Fig. 1c) to fully utilize the benefits of heterogeneous hardware resources, as shown in Fig. 1b. The framework incorporates graph construction to build a compiler-assisted dependency graph (Fig. 1d) and resource-aware optimization (Fig. 1e) to fully utilize the underlying heterogeneous hardware (Fig. 1f). The motivation of our approach is that *function calls in HLLs can be considered as taking inputs from previous blocks and generating outputs to the next blocks*. This is similar to how our brain works in terms of neurons and synapses, which have several fundamental properties^14^. (1) Highly parallel operation: function calls can be potentially operating simultaneously for handling different input arguments like neurons. (2) Interleaving memory and processing: communication and computation usually coexist in a function that performs calculation and stores results. (3) Inherent scalability: the hardware is supposed to be scalable as inserting additional functions in a program requires the connection of the extra hardware pipeline. (4) Event-driven communication: the hardware starts to compute when data stored in queues become available. We evaluate a wide range of real-life software applications such as deep learning, drone controller, autonomous state estimator, and brain-machine interface and validate that the GAHLS framework not only provides a self-optimization to AI systems but also outperforms the state-of-the-art hardware design techniques by 14.27×.

### Problem formulation and framework overview

In order to combine the benefits of highly programmable processing elements (e.g., CPUs, GPUs) with high performance and efficient domain-specific accelerators, as well as to enable programming flexibility for future emerging AI systems, as opposed to the traditional device mapping problem, we formulate a new heterogeneous computing system optimization problem to be considered within the high performance computing, energy-efficient sensing/perception and computing systems, computing systems for machine learning (ML) and artificial intelligence (AI) at the edge and at scale, computational imaging systems, and human-centered signal processing contexts. Therefore, we adopt the message passing communication mechanism  ^15^ to automatically synthesize efficient hardware accelerators. To enable the design of self-reliant, self-programmable and autonomous computing systems paradigm (going beyond von Neumann architectures), this approach first collects dynamic low level virtual machine (LLVM) universal intermediate representation (IR) instructions with annotated function calls from high-level programs (HLPs). In this way, we can understand the data dependencies between universal IR instructions and the relationship between instructions and function calls. Secondly, we represent the LLVM IR instructions through a *compiler-assisted dependency graph* (CaDG) where the nodes represent computations (e.g., addition, subtraction, multiplication) or function calls, and the edges represent data dependencies or direct input dependencies with function calls. Next, we propose a suite of resource-aware optimizations while maximizing the overall performance (i.e., the number of utilized resources does not exceed a hardware/system-related constraint or user-defined upper bound). These optimization steps include similar subgraph detection by partitioning the CaDG into several similar clusters, common feature extraction by analyzing the common patterns among clusters, and aggregation by merging similar clusters into one to reduce the number of required resources. Finally, we synthesize the CaDG into heterogeneous accelerators. There are two types of hardware nodes, namely, the control and computation nodes. The control nodes mimic the mechanism of function calls by receiving data from previous nodes (similarly to the way arguments work) and route data to the correct output ports. The computation nodes, translated from subgraphs, calculate data from upstream nodes and send it to the next nodes.

### Static and dynamic compilation captures the different complex graph motifs in a CaDG

**Figure 2:**
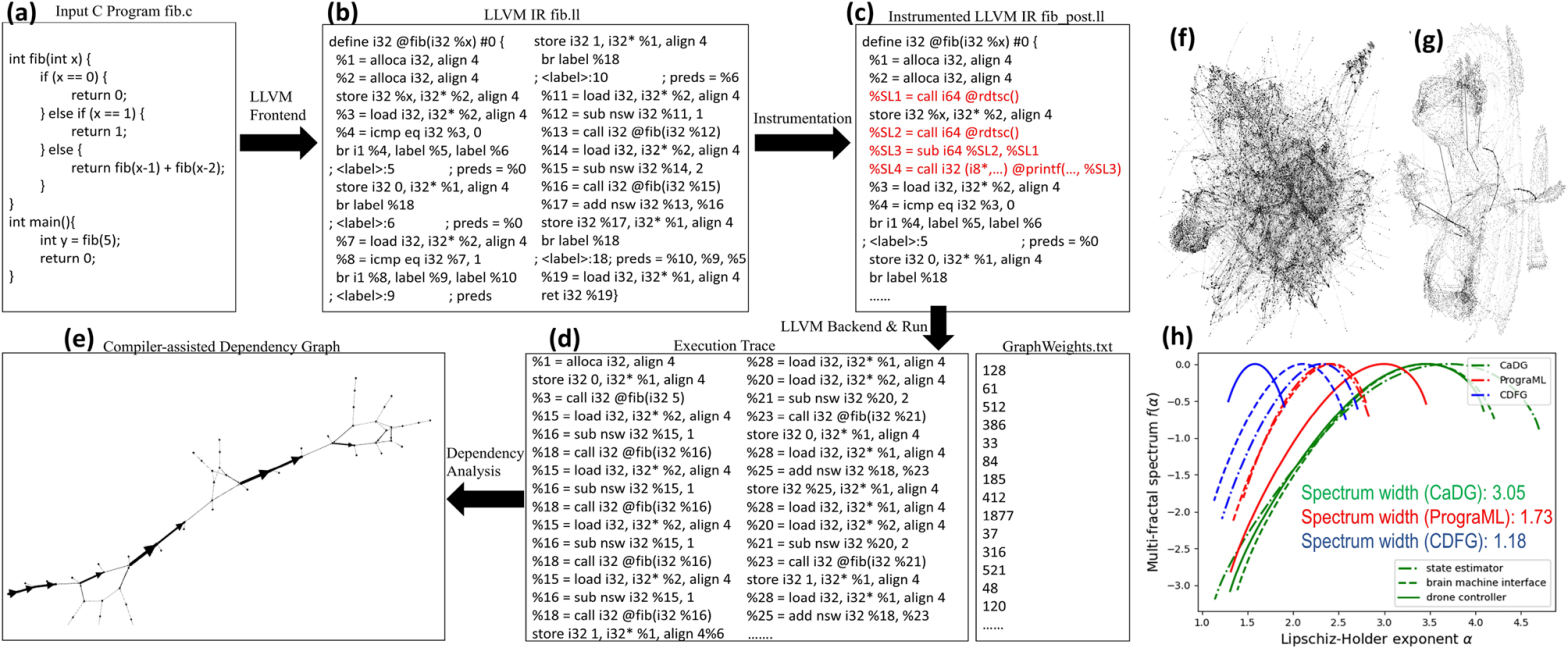
Autonomous dynamic compilation and dependency analysis of high level programs enable the formation of heterogeneous graph structures to be explored and synthesized onto optimized hardware units. (**a**–**e**) The procedures of constructing a CaDG from a software kernel (**a**) that involves static compilation (**b**), code instrumentation (**c**) to get the weights for memory operations, dynamic compilation (**d**) to obtain the execution trace, and dependency analysis (**e**) to construct the graph; (**f**–**g**) real-life CaDGs from two real-life applications, i.e., autonomous state estimator and brain machine interface; (**h**) multifractal spectra for three software applications. The wider the multifractal spectrum of a graph, the more heterogeneous the graph.

Recently, various LLVM IR based frameworks to generate graph representations for the source code were proposed (e.g., PrograML ^16^, abstract syntax tree  ^17^). The graph representation enables machine learning strategies to mine and understand the topological structures of algorithms and computer programs encoded in their graph representations  ^18^. However, such graph representations based on static analysis have a few limitations in parallel computing ^9^. First, memory dependencies are hard to analyze in static analysis as it cannot identify physical memory addresses in *load* and *store* at compile-time. Such unknown dependencies could potentially increase data communication overhead if dependent memory operations are mapped to different cores. Second, the number of iterations in a loop sometimes cannot be statically determined. This unknown information could have a significant impact on loop vectorization ^19^ due to load imbalance. Therefore, rather than performing static compilation, we collect dynamic execution traces with representative input seeds to reflect true dependencies.

Specifically, we first compile the input program (Fig. 2a) into the static LLVM IR via the frontend in Fig. 2b. Next, for each memory instruction, we insert the *rdtsc* function to calculate the time it takes to execute on a universal machine as indicated by the red instructions in Fig. 2c. Lastly, we run the code via the backend to collect the execution trace and timing information in Fig. 2d. In this way, loops are unrolled such that we can achieve high parallelism by synthesizing them in hardware. Besides, due to message passing used in the hardware architecture, we abstract away from shared memory operations such as load and store as *enqueue* and *dequeue* operations to communicate with FIFOs and act as inputs and outputs to connect with the control nodes. Finally, we analyze the data, control, and memory dependencies between instructions to construct a CaDG as shown in Fig. 2e. We also show two CaDGs from real-life applications, namely, autonomous drone state estimator and brain machine interface in Fig. 2f–g. Next, we provide a formal definition of the CaDG.

#### Definition 1

A compiler-assisted dependency graph (CaDG) is defined as a weighted, directed acyclic graph $\mathscr {G}(n_i, e_{ij}, w_{ij}, attr_i| i,j\in \{1,\ldots ,N\}, attr_i\in \mathbb {R}, w_{ij}={dir_{ij}\cdot n_i\cdot n_j\in \{-2N+1, \ldots , 2N\}}, dir_{ij}=\{-1, 1\})$ where each node $n_i$ represents a function call (control node with $n_i=0$) or instructions ($n_i>0$); each edge $e_{ij}$ represents data dependency between nodes *i* and *j*. Each node attribute represents the time it takes to execute. Each weight of an edge $w_{ij}$ depends not only on the types of two connected nodes $n_i$ and $n_j$, but also on the direction of the associated edge.

The CaDG representation exhibits various self-similar structures because of the loops and conditional statements. To quantify the higher-order topological complexity, we perform the multifractal analysis of these graphs and quantify their properties through the multifractal spectrum (Fig. 2d). The width of the multifractal spectrum $f(\alpha )$ with respect to the Lipschitz-Holder exponents $\alpha$ measures the structural complexity and heterogeneity of a network ^20,21^. Here, $\alpha$ quantifies the dimension of the fractal structure, and $f(\alpha )$ reflects the proportion of fractal structures with a given Lipschitz-Holder exponent $\alpha$, i.e., the distribution of fractal structures in the network. The multifractal spectrum of a monofractal graph is similar to a delta function where a single physical rule governs the graph structure at any scale and can be interpreted in terms of the complexity of the system. Therefore, the more topologically complex a CaDG representation, the wider the corresponding spectrum width. In the experiment, we compare CaDG with different graph representations of programs such as PrograML^16^ and control-data flow graph (CDFG)^22^ to understand which method can exploit the heterogeneity of code structures. As we can see from Fig. 2h, the width of the multifractal spectrum from CaDGs is, on average, 3.05, compared to PrograML (1.73) and CDFG (1.18). This demonstrates that the CaDGs constructed from the static and dynamic compiler analysis exhibit more heterogeneous graph motifs to be explored and synthesized onto hardware. Compared to the traditional CDFGs, the CaDG representation offers the following advantages that could facilitate the process of hardware design and optimization: (1) Node representations are different. In the CDFG, nodes represent either control instructions such as function calls and jumps/basic blocks, depending on the actual implementation, or variables/data. However, in the proposed CaDG, the nodes represent actual universal assembly instructions, including computations, which allows to consider a wide variety of computing paradigms and numerous other system optimizations that remain to be discovered by the computer science and computer engineering communities. (2) The structures of the two graphs are different. The CDFG does not have self-similar structures whereas, the CaDG does possess self-similar properties, and this is a key observation that we further utilize for downstream optimization steps targeting higher performance with minimal hardware resources. (3) In CaDGs, loops are unrolled, and we perform alias analysis to address memory dependencies that are hard to decide in CDFGs at compile-time. In this way, granted that usually the CaDGs are bigger than CDFGs, the CaDGs are much more fine-grained, which facilitates us to design an optimization model to partition them into clusters with minimal data communication overhead caused by memory stalls.

There are three types of graph motif representations of the building blocks that can constitute any program in our framework, namely, a tree structure, a star structure, and a mesh structure. A tree structure represents any sequential code that is being executed in LLVM due to dependency analysis in the static single-assignment form. A star structure represents any loops that contain array operations. The LLVM IR “*getelementptr*” is widely used to get the pointer of the first element in an array, which is the center of the node in a star structure as it is used to distribute different elements of the array to different portions of code to be processed. A mesh structure represents any loops without arrays. It is similar to a pipeline where one iteration of work has to finish before the next iteration starts.

**Figure 3:**
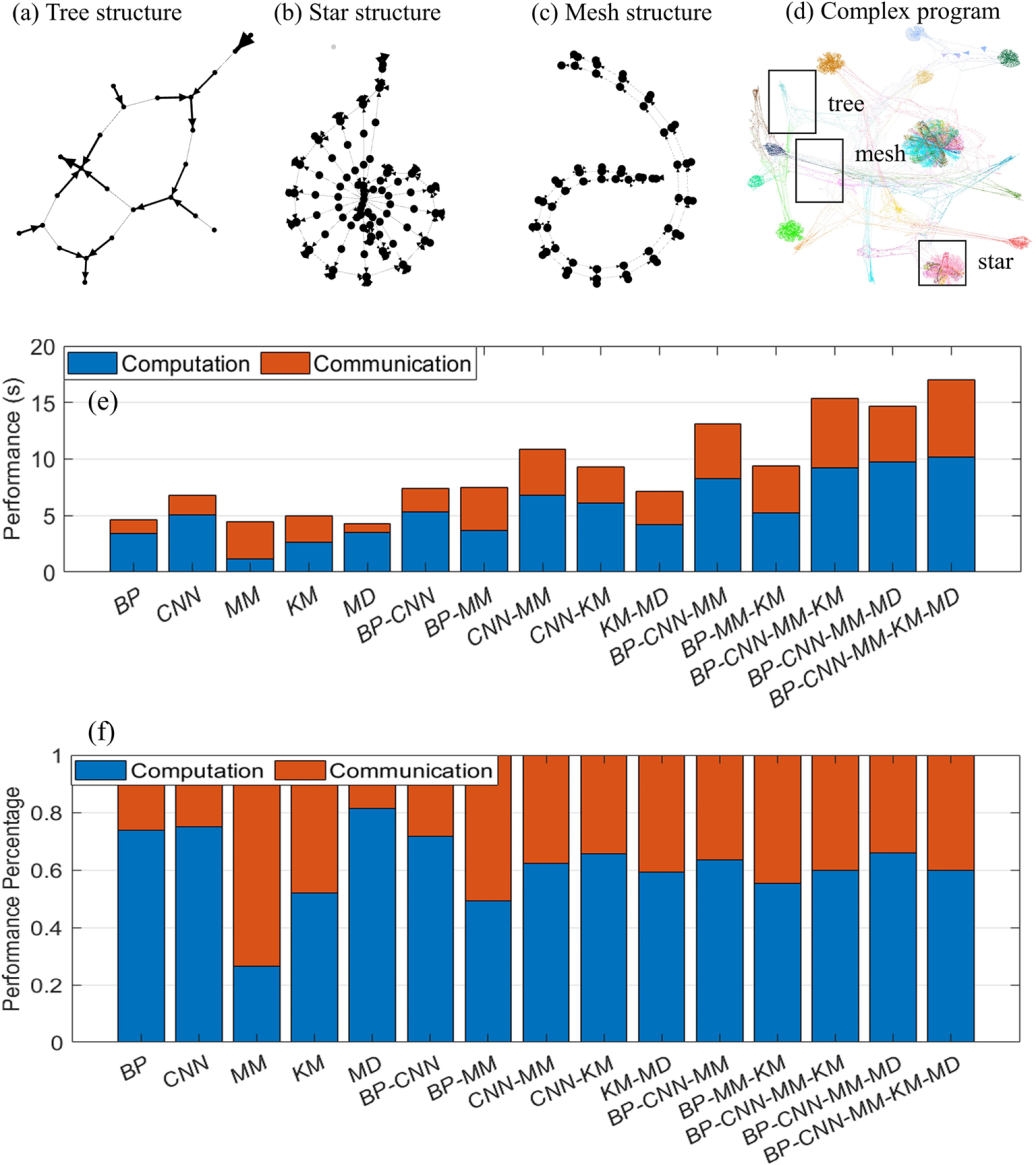
Graph motifs within computer programs (software). (**a**) A tree-like motif corresponding to a small code consisting of sequential code. (**b**) A star-like motif corresponding to a small code consisting of a *for* loop with arrays. (**c**) A mesh-like motif corresponding to a small code consisting of a *for* loop without arrays. (**d**) A complex (software) program exemplifying the existence of many tree-, star-, and mesh-like structural motifs. (**e**) By running various combinations of complex programs, we can measure and compare the time spent on computation and communication for each combination of considered programs. (**f**) For the same setup as in (**e**), we can compare the percentages of time spent in either the computation or the communication obtained by exploiting the GAHLS design methodology. We can observe that by running multiple programs at the same time does not increase the computation significantly whereas the communication time increases somewhat due to waiting periods caused by memory transactions. Therefore, by running the combination of all programs at the same time is 1.48$\times$ faster compared to running each individual program in a sequence.

Each smaller program can be transformed into a graph that has as graph motifs one of the three graph representations exemplified in Fig. 3a–c. Therefore, different smaller programs can constitute a large complex program. As we can see from Fig. 3d, the color of the nodes reflects a building block, and our framework can handle a variety of programs and transform them into a large complex graph with several subgraph motifs. The synthesizer then can analyze the graph and then optimize the graph while taking into account the available resources. Finally, it generates the ideal hardware in which the processing nodes and control nodes are interconnected like in network-on-chip architectures^23^ to provide efficient data communication.

In the end, the architecture generated by our framework depends on the topological structures of the input programs. The architecture is a hybrid of tree, star, and mesh structures that can be efficiently run in parallel.To examplify our design methodology, we choose a variety of high level programs such as matrix multiplication (MM), backpropagation (BP), and convolutional neural networks (CNNs) that are commonly encountered in many machine learning and artificial intelligence architectures to perform an ablation study and run combinations of these programs to demonstrate how can this proposed framework handle complex programs that concomitantly depend on these classical ML algorithms. As we can see from Fig. 3e–f, running multiple programs at the same time does not increase the computation a lot as most of the portions are designed to be executed in parallel whereas communication time increases somewhat to wait for memory transactions between cores and memories. For example, for running the combination of all MM, KM, MD, BP, and CNN programs at the same time, the performance is 17.0 s, which is 1.48$\times$ faster compared to running each individual program in a sequence.

### Heterogeneous emerging memory technology enables high bandwidth and fast data communication

**Figure 4:**
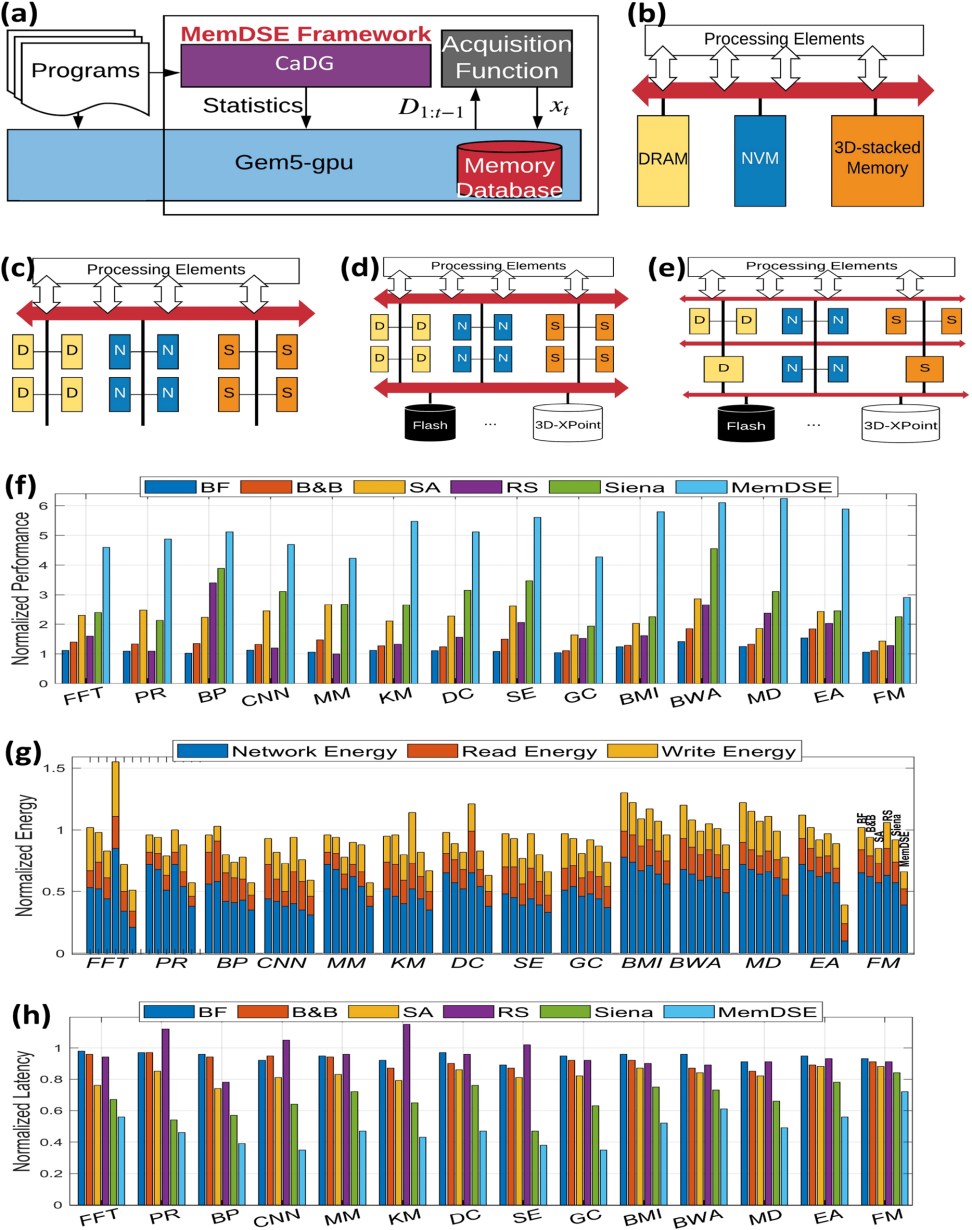
Overview of the MemDSE framework. (**a**) MemDSE is similar to a wrapper function whose goal is to explore the memory design space. It collects the noisy outcome statistics into data from gem5-gpu and generates a new architecture by optimizing the acquisition function. In addition, statistics collected from program abstraction help the search into possible regions of a memory architecture to improve the overall performance. (**b**–**e**) Memory architectures: (**b**) centralized memory, (**c**) distributed memory, (**d**) persistent memory and distributed memory, (**e**) and different sizes of memory (heterogeneity). (**f**–**h**) We compare MemDSE with different search algorithms in terms of the normalized speedup (**f**), energy (**g**), and latency (**h**). The experimental results indicate that MemDSE outperforms the rest by at most 6.23$\times$.

Graph analytics, machine learning, AI, big data and complex data science applications call for high performance computing which consists of processors and memory to provide real-time analysis, high performance, and efficient energy consumption. While the shrinking of transistor geometries has led to fast computation in processors, the memory system remains as a fundamental performance and energy bottleneck in all computing platforms. Therefore, heterogeneous memory systems^24–28^ are proposed to include multiple different technologies or multiple different types of the same technology with different properties, e.g., density, bandwidth, cost, speed, latency, power: non-volatile memory (NVM), such as 3D-XPoint^29^, phase-change memory (PCM)^30^, and spin-transfer-torque magnetic random access memory (STT-MRAM)^31^; and high-performance volatile memory, like hybrid memory cube (HMC)^32^, wide-I/O 2 (WIO2)^33^, high-bandwidth memory (HBM)^34^, and graphics double data rate 6 (GDDR6)^35^. A major question to address is how to find a specific hybrid memory architecture to provide high memory bandwidth and efficient energy consumption for each program. For example, big-data memory intensive programs significantly benefit from an efficient 3D-stacked memory technology that offers high bandwidth whereas it is not necessary for a simple program with tens of lines. The goal is to optimize a cost function over the possible space of memory architectures, which is hard to evaluate and does not have a closed-form expression. Therefore, to further exemplify the benefits and capabilities of the GAHLS design methodology, we develop the memory design space exploration (MemDSE) framework capable to exploit the GAHLS formalism to select and design efficient heterogeneous memory systems based on Bayesian optimization for global optimization of the black-box cost function. We further consider the computational properties in the CaDGs to explore the space of memory architectures.

Designing hybrid memory architectures without considering the program properties cannot effectively tackle the memory wall. Therefore, we collect statistics used to describe the execution and behavior from the CaDG. We apply these statistics as extra features for exploration and exploitation of the memory architecture. For example, the function should explore frequently the region of 3D-stacked memory for graph processing workloads as this memory technology can provide high bandwidth to overcome the limitations of random memory accesses. Therefore, we collect the following statistics: the number of computations (*cmpt*), the number of memory read and write operations (*read*/*write*), the number of edges, the shortest/average/longest path lengths (*spl*/*apl*/*lpl*). In addition, the CaDG also captures the underlying data allocation and layout. It is important to decide data allocation in the heterogeneous memory design as it is dependent on where and how data are fetched. We partition the data into different clusters to minimize data communication between clusters ^9^ and map each cluster to an appropriate technology to maximize the reward. For example, a cluster with more than hundreds of write or read operations is beneficial to be mapped onto high-bandwidth memory such as HBM and HMC.

In the evaluation, we compare the proposed MemDSE for different search strategies: brute-force (BF), branch-and-bound (B&B), simulated annealing (SA), random search (RS), and state-of-the-art technique called Siena ^36^. BF enumerates and searches all of the possible outcomes at the expense of a large required computational time. In the implementation, we set an upper time limit for BF. B &B explores branches of a set of candidate solutions. Before enumerating the possible outcomes of a branch, the branch is checked against upper and lower estimated bounds on the optimal solution, and is discarded if it cannot produce a better solution. For the brute-force and branch-and-bound strategies, we also make sure that the maximum depth of memory architectures is 3 to constrain the number of possible states to search within a reasonably meaningful time limit. SA is a heuristic search algorithm to approximate the global optimum of a given function ^37^. In the implementation, we use performance$\times$energy$\times$bandwidth as the cost function for SA to explore and an exponential cooling scheme, i.e., $T_{k+1}=0.95T_k$. RS randomly explores a possible branch and accepts it if it is better. Siena ^36^ is the state-of-the-art technique to explore the organization of heterogeneous memory systems. We use 14 real-life applications to evaluate the effectiveness of MemDSE that includes fast Fourier transfer (FFT), pagerank (PR), backpropagation (BP), convolutional neural network (CNN), matrix multiplication (MM), k-means (KM), drone controller (DC), state estimator (SE), Granger causality (GC), brain machine interface (BMI), Burrow-Wheeler aligner (BWA) for DNA alignment, molecular dynamics (MD), evolutionary algorithm (EA), and Fiduccia-Mattheyses (FM) hypergraph partitioning.

As we can see from Fig. 4, in terms of performance improvement, MemDSE provides 2.17$\times$ and 6.22$\times$ higher speedup compared to the state-of-the-art technique Siena and different search strategies, respectively. In terms of energy consumption, occasionally some applications consume higher energy compared to the default DRAM technology. For example, energy consumption of the FFT with BF and RS is 1.02$\times$ and 1.57$\times$ worse, respectively. In general, MemDSE offers lower network energy for data movement due to the fact that MemDSE constructs the heterogeneous memory topology. It allows data to bypass the highly congested interconnects and reduce the number of hops from the source to the destination, leading to 2.19$\times$ and 2.37$\times$ higher improvement compared to Siena and baseline. For the normalized average latency, memory architectures generated by MemDSE can have at most 2.63$\times$ improvement. This is achieved by minimal data communication and heterogeneous memory topology to improve the number of hops from the source to the final destination.

### Resource-aware optimization helps to determine the optimal resources to improve application performance

**Figure 5:**
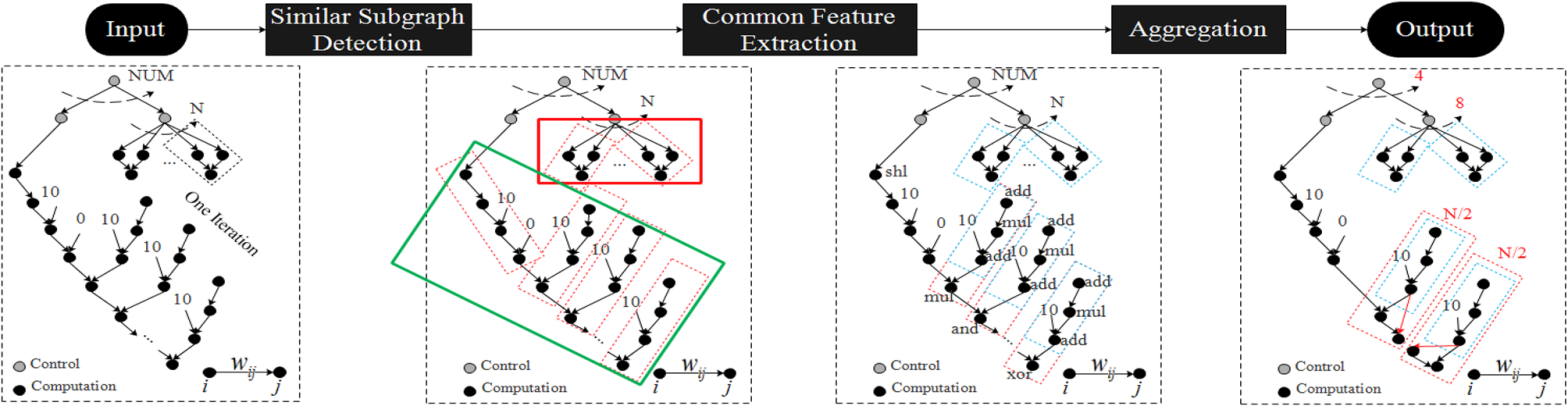
Resource-aware optimization steps in GAHLS: similar subgraph detection, common feature extraction, and aggregation. We first partition the CaDG into different sets of similar subgraphs as indicated by the red dotted boxes. Next, we extract common features from subgraphs using the suffix tree, shown in the blue dotted boxes. Finally, we merge common structures to reduce resource usage.

In case there are a large number of iterations in the loops to be unrolled, the synthesized hardware accelerators may exceed the maximum available resources such as LUTs, memories, and IOs that a chip can provide. Therefore, due to limited resources, it is crucial to ensure that resources utilized in programming the hardware accelerators are within a user-defined bound while maximizing the system performance. Figure 5 illustrates our proposed three-step resource-aware optimization, which consists of (1) self-similar subgraph detection which seeks to partition a large CaDG into several similar subgraphs; (2) common feature extraction which seeks to determine the intersection of common graph motifs among these subgraphs; (3) an aggregation step which merges several subgraphs with the same structure into a bigger graph while exploiting their commonalities for reducing resource utilization and optimizing the performance profile.

More specifically, once the common structures are identified from subgraphs, we then combine the same structure such that we fully utilize the user-defined upper bound of resources while achieving high parallelism. Therefore, we propose an integer linear programming (ILP) based optimization model to balance resources and parallelism due to a trade-off between merging the common structure of different subgraphs to reduce resource utilization and parallel system performance. The model is parameterized by three parameters *a*, *b*,  and *c* (0–10) that control look-up tables (LUTs), block memories (BRAMs), and IOs, respectively. The higher a parameter, the higher the corresponding resource utilization. We perform the ablation study to see how each parameter contributes to the resource utilization and runtime speedup. As we can see, maximizing the resource utilization ($a=b=c=10$) could in general improve the runtime speedup over 13.53$\times$ on average while resource utilization is high. To slightly reduce the resource utilization ($a=b=c=7$) could still provide on average 9.34$\times$ performance improvement in a resource-constraint platform.

**Figure 6:**
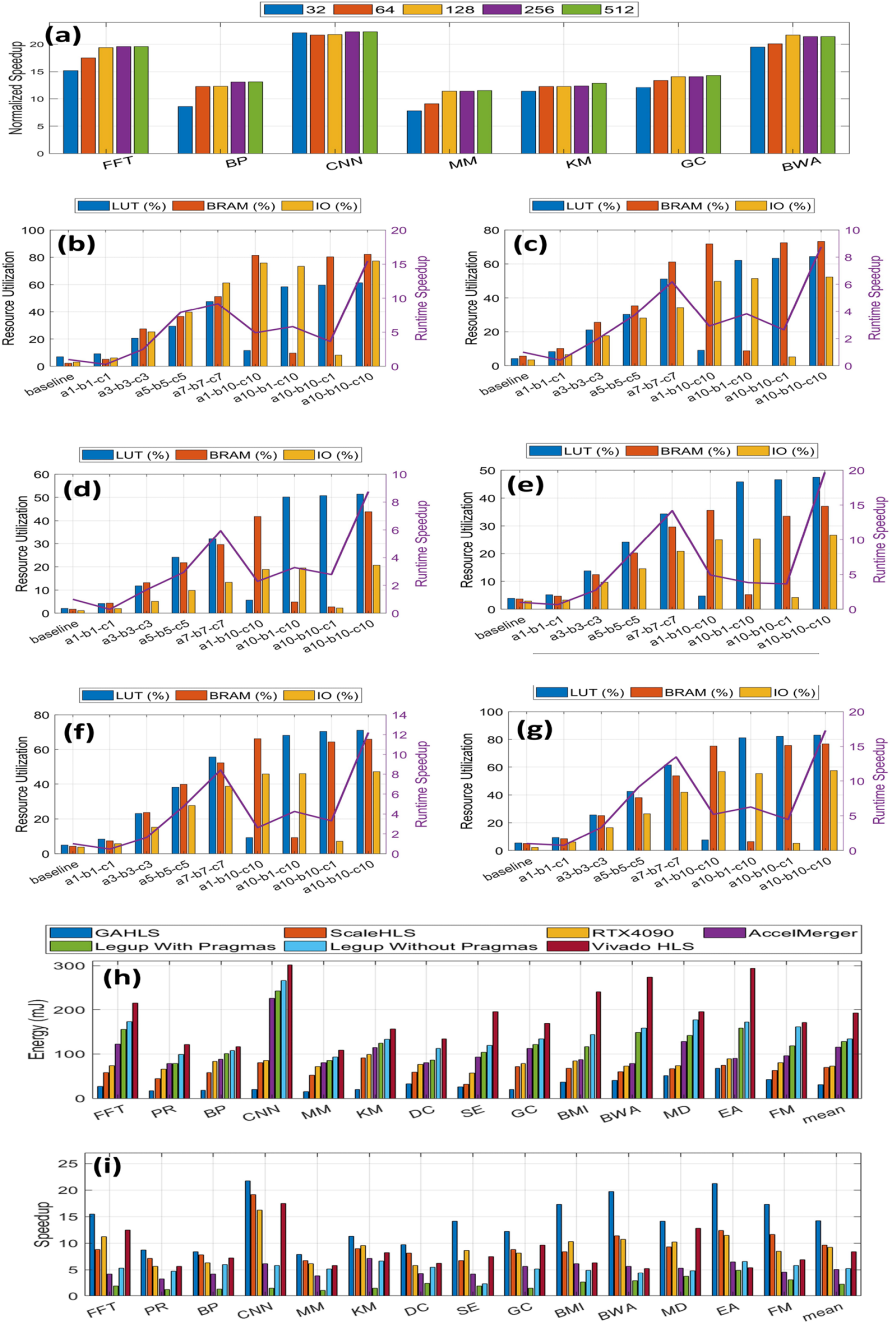
Framework comparison among GAHLS, state-of-the-art ScaleHLS, and some well-known HLS tools such as LegUp and Vivado HLS. (**a**) We perform scalability analysis of GAHLS on some applications with the problem size scaled from 32 to 512. (**b**–**g**). Ablation study on the ILP based optimization in the aggregation step to see how the parameters control resource utilization and have an impact on runtime speedup for (**b**) FFT, (**c**) PR, (**d**) BP, (**e**) BWA, (**f**) GC, and (**g**) BMI. (**h**–**i**) We compare GAHLS with some state-of-the-art techniques in the field to validate the effectiveness of our framework. in terms of speedup (**h**) and energy (**i**).

We then perform scalability analysis to understand how the GAHLS framework performs on different problem sizes by considering six benchmarks from 32 to 512. As shown in Fig. 6a, for some compute-intensive applications such as CNN and KM, GAHLS can reliably provide consistent speedup for all of the problem sizes. For the rest of applications that are less compute-intensive, they require less resources for a small problem size, leading to a less efficient design. Increasing the problem size helps GAHLS fully utilize the available on-chip resources to maximize the speedup. Nevertheless, due to resource-aware optimization, GAHLS is able to provide high speedup by wisely utilizing the available resources.

We further compare GAHLS with several state-of-the-art techniques such as ScaleHLS^38^, Vivado HLS^39^, and LegUp^40^ as shown in Table 1 and Fig. 6h–i in terms of performance and energy consumption. Of note, in contrast to GALHS which is fully autonomous, the ScaleHLS, Vivado HLS and LegUp require significant additional programming effort from a hardware designer (i.e., inserting pragmas at specific places on the high level programs) to provide high performance results. All performance improvements in the evaluation are then normalized with respect to LegUp without pragma. For all the compute-intensive applications, the performance improvement of the GAHLS framework over LegUp with pragmas ranges from 1.92$\times$ (for BP) to 4.07$\times$ (for CNN), over LegUp without pragmas ranges from 8.11$\times$ (for MM) to 21.71$\times$ (for CNN), and over ScaleHLS ranges between 1.08$\times$ (for BP) and 2.10$\times$ (for SE). The proposed GAHLS framework achieves this speedup because it better characterizes the topological properties of various highly interdependent high-level programs, identifies the parallelism, and tailors the processing architecture and its hardware utilization to the actual structural requirements encoded in the CaDG. Table 1 illustrates the number of resources in GAHLS, ScaleHLS, LegUp with pragmas, and Vivado HLS. GAHLS can fully utilize the underlying hardware resources (LUTs, BRAMs, and IOs), thus it achieves the best speedup. However, in some cases where the area should be as small as possible such as a resource-limited embedded platform, we can fine-tune the values of *a*, *b*, *c* to control the number of hardware resources to be used in Fig. 6b–g. In addition, we also compare GAHLS with AccelMerger^41^ and RTX4090. AccelMerger is not able to produce better performance compared to GAHLS as it cannot efficiently utilize the underlying hardware. On the other hand, we tested different benchmarks running on RTX4090 GPU in terms of performance and energy consumption. For some benchmarks that inherently possess high degree of parallelism such as MD and CNN, the modern GPU can achieve almost the same performance as GAHLS but for the rest that are hard to parallelize or involve a lot of memory manipulations, GPU is not as efficient as FPGA due to dominating sequential execution and data communication that suffer from the highly parallel architecture provided by GPU.

**Table 1:** Resource utilization comparison.

	GAHLS ($a=b=c=10$)				ScaleHLS (G7+L7+D)				LegUp with pragmas				Vivado HLS			
	LUTs	BRAMs	IOs	Fmax	LUTs	BRAMs	IOs	Fmax	LUTs	BRAMs	IOs	Fmax	LUTs	BRAMs	IOs	Fmax
*FFT*	38,928	3995	162	245	33,151	3254	132	133	17,104	503	52	134	10,218	215	30	207
*PR*	40,830	3558	151	194	30,118	2813	97	154	20,145	323	35	142	12,742	558	20	162
*BP*	32,587	2129	42	177	28,941	2336	58	154	10,258	164	63	147	9248	133	42	176
*CNN*	58,429	3660	180	193	51,282	3180	128	176	34,156	2588	77	117	24,583	2109	48	156
*MM*	35,766	2861	68	136	28,471	1768	82	114	16,593	124	39	88	15,479	110	21	121
*KM*	49,542	2295	31	155	38,602	1652	42	144	23,378	135	46	151	16,385	118	33	189
*DC*	52,829	3960	188	174	42,658	3416	142	152	19,512	2166	54	140	16,775	1884	54	146
*SE*	46,135	2526	136	146	39,382	2280	144	128	22,856	1658	44	154	20,088	965	44	166
*GC*	45,077	3203	99	138	33,827	2742	86	120	27,611	2162	50	144	24,588	2204	50	150
*BMI*	52,685	3732	121	138	38,284	3180	106	124	25,660	2890	50	170	26,854	2881	50	164
*BWA*	30,115	1803	56	134	27,568	1637	44	122	19,512	2577	54	140	16,775	3118	54	146
*MD*	48,461	2658	144	158	36,952	2286	122	130	22,856	1630	44	154	20,088	782	44	166
*EA*	32,926	1878	96	172	28,186	1422	68	154	27,611	2658	50	144	24,588	2869	50	150
*FM*	28,120	1418	68	162	21,583	1228	48	148	25,660	2165	50	170	26,854	2277	50	164

We compare resource utilization of different existing HLS tools with GAHLS. As we can see that compared to LegUp and Vivado, GAHLS can fully exploit the underlying hardware, which can be also adjusted by hypeparameters.

**Figure 7:**
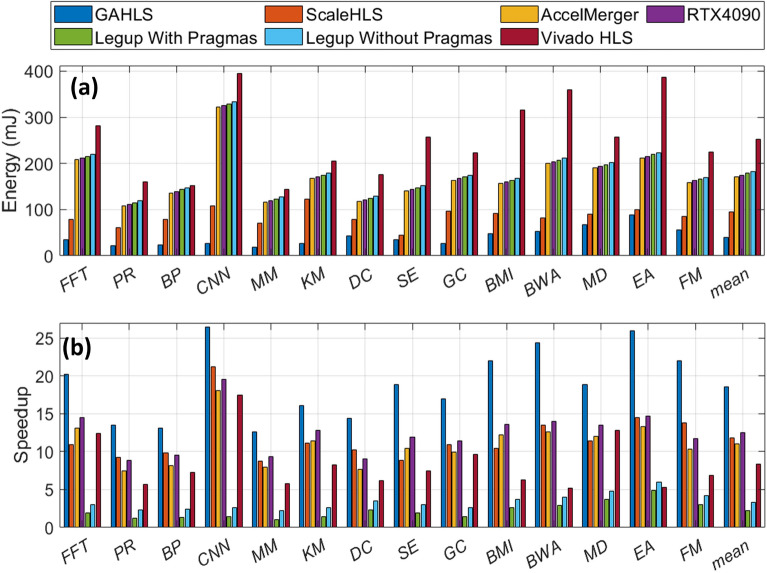
Performance (**a**) and energy (**b**) comparison among GAHLS, state-of-the-art ScaleHLS, and some well-known HLS tools such as LegUp and Vivado HLS.

In addition, our proposed framework is applicable to large-scale hardware devices as well. The rationale for broad applicability of our approach is justified by the fact that it exploits a novel efficient resource-aware optimization summarized in the Methods section. The key idea in this optimization strategy focusing on identifying the optimal hardware resources with respect to targeted performance and satisfying desired constraints is to analyze, identify common topological motifs hidden in the induced topology corresponding to the algorithms, and combine the identified common topological structures from the graph representation of essential computations and communications of the algorithm and code into a compact topological motif while taking into account the user-defined upper bound of resources and achieving a high degree of parallelism. Therefore, this novel optimization model balances the required resources and maximizes parallelism by exploiting a trade-off between merging the common structure of different subgraphs, reducing the resource utilization and maximizing parallel system performance. Hence, the framework can handle both small-scale and large-scale hardware platforms. Below you can find application performance and energy consumption on a large-scale hardware platform. Specifically, we are using Xilinx Alveo U200 which has 1182K LUTs, 2364K registers, and 6840 DSP slices, which we received permission to use from another academic group because we lack the financial resources to purchase it. We set the hyper-parameters a, b, and c all to be 1 to fully utilize the underlying resources. We believe, as shown in Fig. 7, our framework is more suitable for a large-scale hardware platform because the underlying resources are fully utilized for different applications. Application performance improvement is 2.10$\times$ compared to ScaleHLS and 5.49$\times$ compared to Vivado HLS. In addition, the energy consumption improvement is 4.61$\times$ compared to ScaleHLS, 15.04$\times$ compared to Vivado HLS, and 26.44$\times$ compared to LegUp, respectively.

To enable the design of the intelligence required in future autonomous AI systems capable of exploiting current and emerging computing technologies and reconfiguring the computing platform as a function of the sensing, communication, computation and actuation requirements, we present a graph analytics based framework to extract and mine the topological and geometric characteristics of high-level programs while assuming a universal Turing machine (that is not constrained to a specific memory model and memory hierarchy), identify various degrees of parallelization as a function of available or desired heterogeneous hardware resources, and better design of the hardware accelerators. First, we transform the high level programs written in C/C++ languages to dynamic LLVM IR instructions through a comprehensive static and dynamic compiler analysis. We use this universal machine IR to track the computational and communication interdependencies to construct a general and flexible CaDG representation where nodes represent computations or function calls and edges represent data dependencies. Second, we propose a three-step resource-aware topological optimization approach to reduce resource utilization and consisting of similar subgraph detection, common feature extraction, and aggregation. Our evaluation with several real-world benchmarks using Nexys 4 DDR demonstrates that the GAHLS improves performance by at $17.3\times$ and $21.41\times$ compared to LegUp and CPUs execution.

In contrast to the above-mentioned research efforts, the proposed GAHLS framework overcomes the drawbacks of state-of-the-art approaches and makes the following novel contributions: Firstly, we avoid the burden put on programmers to insert pragmas into HLLs by adopting a ***compiler analysis*** that translates the HLPs into a universal LLVM IR. Secondly, the proposed framework constructs a CaDG to extract and represent only the meaningful computation and communication operations of interacting and highly interdependent HLPs. Next, the GAHLS framework provides ***analytical tools to mine the topological complexity of CaDG*** and identify the most efficient design of a parallel set of hardware accelerators. Thirdly, the proposed framework exploits a *resource constrained optimization* approach to map the compact representation of the optimized CaDG onto FPGA resources for an improved degree of parallelism and performance, but without violating hardware constraints.

The benefits, novel contributions and improvements of our proposed framework are as follows:

1. The framework does not rely on using pragmas that must be inserted by an experienced programmer. In contrast, our framework automatically analyzes and parallelizes the target code without the need for tedious offline analysis and burdens on expert programmers for performing these steps without a comprehensive mathematical strategy in mind. In short, anyone in the broad fields of robotics, AI, ML, bioengineering, civil engineering, biomedicine, industrial engineering, aerospace and naval engineering, etc., can use our framework even though she/he does not have any hardware and HLS background knowledge because no insertion of pragmas or proficient knowledge are required; more precisely, no optimization efforts from the programmer are required. Our framework can perform and generate RTL code automatically and with significantly higher performance than all existing approaches which unfortunately require the intervention of expert programmers for inserting specific pragmas and other engineering efforts (e.g., although a proficient programmer can have significant experience with pragma insertion, his prior experience becomes useless when faced with a new code that has just been released and for which we lack knowledge on the optimal parallelization; our framework addresses this as well through the above mentioned comprehensive optimization strategy).
2. The optimal memory architecture and selection have been identified to achieve the best memory bandwidth and provide the most efficient communication. Without considering these aspects, the memory wall and communication challenges are major roadblocks for the entire field of designing extremely large heterogeneous multiprocessor systems-on-chip for edge, fog and data-center computing.
3. The loops are unrolled fully to a point where the underlying resource utilization is fully utilized. Users have the option and ability to adjust what percentage of hardware resources is desired. It opens new design methodologies and directions for future engineering of computing platforms running from underwater, to terrestrial and space applications (all of those have specific strict energy-delay/power-performance envelopes). We performed an ablation case study and presented in Fig. 6b–g considering different optimization hyperparameters that have impact on resource utilization and system performance.

In what follows, we provide an idea of extending the current framework to support existing popular deep-learning frameworks such as PyTorch and Tensorflow using MLIR. Given the current advances in machine learning, the MLIR refers to a compilation framework that supports multiple levels of functional and representational hierarchy. MLIR includes a single static assignment (SSA) style IR where an Operation is the minimal unit of code. Each operation accepts a set of typed Operands and produces a set of typed Results. Connections between the results of one operation and the operands of another operation describe the SSA-style flow of LLVM. A sequential list of operations without control flow is defined as a Block and a control flow graph (CFG) of blocks is organized into a Region in MLIR. A Dialect in MLIR defines a namespace for a group of related operations, attributes, and types. MLIR not only provides multiple built-in dialects to represent common functionalities, but also features an open infrastructure allowing to define new dialects at different abstraction levels.

Pass is a key component of this new compiler approach which traverses the IR for the purpose of optimization or analysis. We can use existing third-party front-ends, Torch-MLIR and ONNX-MLIR, to parse PyTorch and ONNX models, respectively. Torch-MLIR first translates PyTorch models into torch dialect, then lowers the IR to affine dialect as the end of compilation. Once dialects are collected from front-ends, we can transform dialects into graph-level IRs and use GAHLS to represent computation graphs.

**Figure 8:**
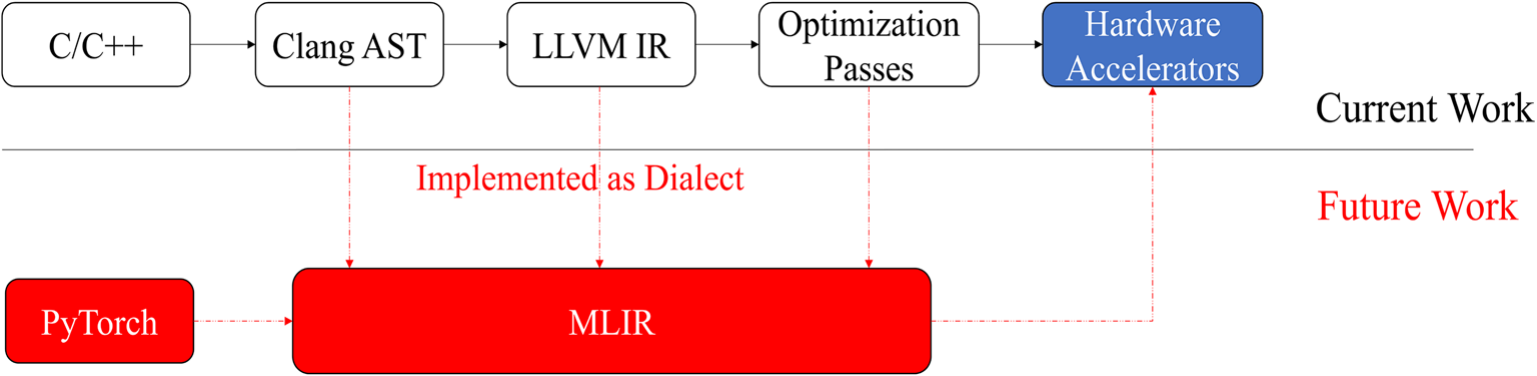
Future work that supports Tensorflow and PyTorch. The idea is to replace different phases of LLVM such as Clang AST, LLVM IR, and optimization passes with Dialects in MLIR. This helps to integrate the whole framework that consists of many components into a unified system that involves different front-ends to support existing popular deep-learning frameworks.

In essence, the idea is to incorporate MLIR into our proposed framework to support Dialects and Operations instead of different phases of LLVM. Then MLIR has different front-end tools to support Tensorflow and PyTorch, as shown in Fig. 8.

In addition, GAHLS can handle application-specific integrated circuit (ASIC) design, as well. More precisely, given the RTL code generated via a GAHLS approach, we can further synthesize it using the semi-custom flow (e.g., using the Design Compiler for logic synthesis and then exploiting the Automatic Place and Route (APR)) using the Cadence Encounter while also considering a set of constraints reflecting the timing and power desiderata. We leave all these extensions for future work while also taking into account the emerging algorithmic developments.

**Datasets.** To study the benefits of the proposed framework, we consider 14 benchmarks representative of recent real-world applications. These benchmarks consist of both simple and nested loops required for perception, inference and decision making in autonomous systems. (1) FFT: Fast Fourier transform with an input vector of size 8092; (2) PR: PageRank is computed iteratively by fetching required data structures from neighboring websites for each website, performing the arithmetic reduction operation described by the core algorithm, updating scores after each iteration. The input file to PageRank is a graph containing 600 nodes; (3) BP: backpropagation used in neural network for back propagating the error to adjust the weights of a neural network. The neural network used in backpropagation has 5 layers of size 20-10-10-10-1; (4) CNN: Convolutional neural network that contains six layers (i.e., CONV, RELU, CONV, RELU, POOLING and fully connected layers) for classifying 64$\times$64 images with 6 feature maps of shape 58$\times$58. (5) MM: Matrix multiplication based on Strassen’s algorithm. The matrices used in experimental results are of size 256$\times$256. (6) KM: K-means clustering with 600 points; (7) DC: Drone controller that represents the high-level program stabilizing and controlling the flight of an autonomous drone when encountering turbulent flow; (8) SE: State estimator that estimates state values in systems with process noise and measurement noise; (9) GC: Granger causality^42^ that determines whether one time series is useful in forecasting another; (10) BMI: Brain machine interface that represents a program for mining the brain activity in actions, decoding the brain state and its evolution, and predicting the human intent^4^; (11) BWA: Burrow-Wheeler Aligner^43^ for short-read DNA alignment; (12) MD: Molecular dynamics for simulating and analyzing the physical movements of atoms and molecules; (13) EA: Lamarckian evolutionary algorithm for molecular design and optimization; (14) FM: Fiduccia-Mattheyses algorithm that solves the hypergraph bipartitioning problem in a heuristic fashion.

**Hardware platform.** For comparison purposes, we use the Vivado 15.2 to generate Verilog implementations corresponding to the considered benchmarks and target the Nexys 4 DDR XC7A100T-1CSG324C as an implementation platform. This platform contains 63, 400 Slice LUTs, 4860 Kbits of fast block RAM, 210 IOs, and 240 DSP slices, and the maximum clock frequency is 450 MHz. The baseline Verilog is generated by an HLS tool, LegUp 6.2^13^ and Xilinx Vivado HLS.

**Resource-aware optimization** The optimization step involves (1) similar subgraph detection to partition a large CaDG into several similar subgraphs; (2) common feature extraction to determine the intersection of common graph motifs among the generated subgraphs; (3) aggregation to merge the subgraphs with the same structure into a subgraph to reduce resource utilization and optimize the performance.

*Similar subgraph detection.* It is not trivial to extract the common features out of a large CaDG with tens of millions or more nodes and edges. The idea is to partition the graph into subgraphs that maximize the mathematical (topological) motifs from a structural information perspective. We then add a constraint as a regularization term to limit the performance difference between subgraphs. We first introduce the following definitions to clarify our approach.

#### Definition 2

A subgraph motif represents the connectivity of different nodes and the direction of edges, which is quantified as the sum of weights of all edges in a subgraph.

1 $$\begin{aligned} W_k = \sum _{ij\in C_k}{w_{ij}} \end{aligned}$$

where $C_{k}$ is the index for a subgraph; $w_{ij}$ represents the direction and connectivity ($dir_{ij}$) of two nodes $n_i$, $n_j$, as discussed in Definition 1.

#### Definition 3

The analytical performance for a subgraph is defined as the time it takes to execute all instructions. For parallel heterogeneous units, we ensure that the workload imbalance is minimized to prevent other units from waiting for the most time-consuming unit.

2 $$\begin{aligned} T_k = \sum _{i\in C_k}{attr_{i}} \end{aligned}$$

where $attr_{i}$ represents the time it takes to execute the instruction as the node attribute collected during the runtime. We calculate the workload of a subgraph as the duration of the execution of all instructions in the subgraph.

#### Definition 4

The similarity among subgraphs is measured as the difference in the subgraph motifs. If two subgraphs have the same motif, the similarity equals 0.

From the above-mentioned definitions, we formulate the similar subgraph detection as follows: Given a weighted CaDG, find the non-overlapping subgraphs $n_c$ with different features which minimize the following cost function

3 $$\begin{aligned}{} & {} \min _{n_c} \quad S_{n_c}+P_{n_c} \end{aligned}$$

4 $$\begin{aligned}{} & {} S_{n_c} = \frac{1}{W} \sum _{\begin{array}{c} 1\le k\le n_c\\ 1\le k'\le k\\ k \ne k' \end{array}} |W_{k}-W_{k'}| \end{aligned}$$

5 $$\begin{aligned}{} & {} P_{n_c} = \frac{1}{T} \sum _{\begin{array}{c} 1\le k\le n_c\\ 1\le k'\le k\\ k \ne k' \end{array}} |T_{k}-T_{k'}| \end{aligned}$$

where $n_{c}$ is the number of subgraph motifs; *k* and $k'$ represent subgraph indices; $W_k$ is the total sum of edge weights in a subgraph *k*; *W* is the total sum of edge weights within the CaDG graph, i.e., $\sum _{i\in G}{W_{i}}$; $T_k$ calculates the execution time in a subgraph whereas *T* represents the total execution time of the entire graph. $W_k$ and $T_k$ vary from each subgraph to another, which are functions of subgraphs.

Intuitively, $W_k$ is a subgraph motif calculated from the weighted adjacency matrix within a subgraph *k*. It represents the types, structures, and connectivity of nodes. We calculate the absolute difference between two different subgraphs *k* and $k'$ (i.e., $\sum _{kk'}|W_{k}-W_{k'}|$) to measure the difference between these subgraphs in terms of structures and edge directions. A smaller value indicates that the subgraphs after partitioning are more similar. In addition, *P* measures the difference between workloads of two subgraphs. In parallel heterogeneous units, it is ideal to balance workloads among tasks to prevent the waiting of other tasks.

However, as we can see later, this problem is NP-hard (Theorem 1), which means that it cannot be solved in polynomial time if $P\ne NP$. Nevertheless, by proving that the function in the equation (3) is submodular and monotonic (Theorems 2 and 3), a greedy algorithm can achieve “good” results (Theorem 4), which can be solved in polynomial time.

#### Theorem 1

The similar subgraph detection problem described in the equation (3) is NP-hard.

#### Proof

Balanced *k*-partitioning problem^44^ finds a set of minimum edges to be removed such that *k* components have equal sizes; it is a known NP-hard problem. This problem reduces to the similar subgraph detection problem in weighted graphs. Therefore, the similar subgraph detection problem is also NP-hard. $\square$

#### Theorem 2

The objective function for the subgraph detection problem is submodular.

#### Proof

Given a CaDG, let us define two different partitions $P^1 = \left\{ s^1_1, \ldots , s^1_n \right\} \subseteq \Omega$ and $P^2 = \left\{ s^2_1, \ldots , s^2_n \right\} \subseteq \Omega$ where $s^i_j$ is the subgraph *j* in the $i-$th partition and $\Omega$ is the set for the solution space for this problem. We define *f*(*P*) as the objective function defined in Eq. (3).

If $$P^1 \cap P^2 = P^c$$, then

6 $$\begin{aligned} \begin{aligned}{}&f \left( P^1\cup P^2 \right) +f \left( P^1\cap P^2 \right) \\&\quad = S|\left( P^1\cup P^2 \right) + S|P^c + P|\left( P^1\cup P^2 \right) + P|P^c\\&\quad = S|\left( P^1 \right) + S|\left( P^2 \right) - S|P^c + S|P^c \\&\qquad + P|\left( P^1 \right) + P|\left( P^2 \right) - P|P^c + P|P^c\\&\quad = \left( S|\left( P^1 \right) + P|\left( P^1 \right) \right) + \left( S|\left( P^2\right) + P|\left( P^2 \right) \right) \\&\quad = f \left( P^1 \right) + f \left( P^2 \right) \end{aligned} \end{aligned}$$

If $$P^1 \cap P^2 = \emptyset$$, then

7 $$\begin{aligned} f \left( P^1\cup P^2\right) +f \left( P^1\cap P^2\right) = f \left( P^1\right) + f \left( P^2\right) \end{aligned}$$

Therefore, by combining Eqs. (2, 3), we can infer that our objective function is submodular because for any two sets $S, T \subseteq \Omega$, $f(S)+f(T)\ge f(S\cap T) + f(S\cup T)$. $\square$

#### Theorem 3

The objective function for the subgraph detection problem is monotonic.

#### Proof

Given a CaDG, let us define the partitions $P = \{s_1, \ldots , s_n\} \subseteq \Omega$ and an arbitrary subgraph $s_k \notin P$.

8 $$\begin{aligned} \begin{aligned}{}&f(P\cup s_k)\\&\quad = \frac{1}{W} \sum _{\begin{array}{c} 1\le k\le n\\ 1\le k'\le k\\ k \ne k' \end{array}} |W_{k}-W_{k'}| + \frac{1}{W} \sum _{i\in P}|W_{s_k}-W_i|\\&\qquad + \frac{1}{T} \sum _{\begin{array}{c} 1\le k\le n\\ 1\le k'\le k\\ k \ne k' \end{array}} |T_{k}-T_{k'}| + \frac{1}{W} \sum _{i\in P}|T_{s_k}-T_i|\\&\quad = f(P) + \frac{1}{W} \sum _{i\in P}|W_{s_k}-W_i|+\frac{1}{T} \sum _{i\in P}|T_{s_k}-T_i| \end{aligned} \end{aligned}$$

Therefore, $$f(P\cup s_k) - f(P) > 0$$. $$\square$$

#### Theorem 4

Given a monotonic submodular function *f*, the greedy maximization algorithm (We can easily convert minimization to maximization in this problem by adding a negative sign to the function.) returns^45^

9 $$\begin{aligned} f(P_{greedy}) \ge \left( 1-\frac{1}{e}\right) \max _{|P|<K} f(P) \end{aligned}$$

where *K* is the maximum number of possible partitions. Therefore, even though the subgraph detection problem is NP-hard^46^, Algorithm 1 is designed to find good enough solutions (partitions). In algorithm 1, we first initialize a partitioning *P* to be a collection of nodes in a CaDG. At lines 5–6, we randomly choose a node *n* from the partitioning and find a partition *P*[*e*] that maximizes the incremental gain in function *f* with respect to node *n*. Then the partition *P*[*e*] is added into the partition where node *n* belongs. The cycle repeats until there are no incremental gains.

**Algorithm 1:**
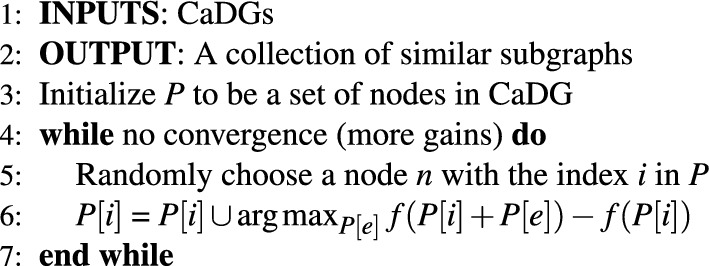
Greedy algorithm for similar subgraph detection.

*Common feature extraction.* With a CaDG partitioned into several subgraphs, we propose an efficient algorithmic approach to extract common features from subgraphs to be merged in order to reduce resource utilization. Before discussing it in detail, we define the meaning of common features.

#### Definition 5

The common feature among a set of graphs is defined as the largest topological structure that is common among these graphs. Some common structures in CaDG could be the star, mesh, and DAG topologies.

A *suffix tree* is a search tree consisting of all suffixes of a given string. The suffix trees provide linear time (efficient) approaches for searches/queries. The time complexity of building a suffix tree from a string of length *V* is $\Theta (V)$. The time complexity of responding to the query of finding a common substring of *A* and *B* is $\Theta (V_i + V_j)$ assuming the length of *A* and *B* is $V_i$ and $V_j$.

We propose a suffix tree based algorithm mentioned in the appendix to find the largest common feature among the subgraphs. It first uses topological sort (TS) for each subgraph to maintain a strict order, which takes $O(V_i+E_i)$, where $V_i$ and $E_i$ are the number of nodes and edges in subgraph *i*. Then, we first build a suffix tree out of the first subgraph, and for the next subgraph, traverse this tree to find the longest path representing the common feature, which takes $O(E_1+E_2)$. We repeat this process until the queue is empty. Therefore, the total time complexity for our feature extraction algorithm is $O(\sum _{i}{(V_i+E_i)})$, which is constrained by the TS algorithm.

*Aggregation.* Once the common structures are identified from subgraphs, we then combine the same structure such that we fully utilize the user-defined upper bound of resources while achieving high parallelism. In this section, we propose another optimization model to balance resources and parallelism due to a trade-off between merging the common structure of different subgraphs to reduce resource utilization and parallel system performance.

The goal is to maximize the system performance while constraining the number of resources used in the final hardware. Therefore, the optimization problem can be formulated as an integer linear program (ILP) problem: Given the CaDG and the associated common features, **find** the right number of resources to be merged in order to

$$\begin{aligned} \begin{array}{ll@{}ll} \max \limits _{n_i} &{} \displaystyle \frac{W}{W_{ser}+\sum _{i}{\frac{W_{par_i}}{n_i}}} &{} &{} (10)\\ \\ {{\textbf {s.t.}}}&{} W = W_{ser} + \sum _{i}{W_{par_i}} &{} &{} (11)\\ &{} \sum _{i}{n_ilut_i}< \frac{a}{10}(LUT-LUT_{ser})&{} &{} (12)\\ &{} \sum _{i}{n_ibram_i}< \frac{b}{10}(BRAM - BRAM_{ser}) &{} &{} (13)\\ &{} \sum _{i}{n_iio_i} < \frac{c}{10}(IO-IO_{ser}) &{} &{} (15) \end{array} \end{aligned}$$

where *W* is the total amount of work generated by the data engine; $W_{ser}$ is the amount of work executed in the serial portion; $W_{par_i}$ is the amount of work executed in the parallel portion *i*; $n_i$ is the number of iterations to be unrolled; $lut_i$, $bram_i$, $io_i$ are the number of LUTs, BRAMs, and IOs used in the hardware accelerator respectively; *LUT*, *BRAM*, *IO* are the total number of LUTs, BRAMs, and IOs available on a specific FPGA; $\alpha$, *a*, *b*, *c* are user-defined parameters to control how many resources should be utilized. In experimental results, we set all of the parameters to 1, which implies that hardware resources should be fully utilized to maximize the parallelism.

After extracting the common features from the similar subgraphs, we can think of the processed CaDG graph as a collection of interconnected subgraphs with common features where nodes represent the common features, i.e., a set of IR instructions in a CaDG with the same structures. Therefore, $W_{par_i}$ is defined as the number of IR instructions within the two or more subgraphs at the same depth after the topological sort. These clusters can be executed in parallel since the input data are available at the same time. Likewise, $W_{ser}$ is defined as the number of IR instructions within only one subgraph at any depth. Because there is only one cluster to be executed when its input data are available, this cluster would count to the serial portion. In addition, this is a general step that can lead to loop parallelism and loop pipelining. It allows us to roll back to the previous scenario to maximize the parallelism. For example, in a resource-scarce situation due to a large number of iterations (say 256), we will roll back t only unroll 16 or 32 loops, leaving the rest pipelined.

We should also reflect this aggregation in hardware by inserting a demux to control the direction of communicating the results. This merge requires a *demux* in hardware to drive the outputs into the correct downstream node. Furthermore, we should perform the credit based flow control by maintaining a counter to signify the downstream nodes when in-coming data are valid.

**HDL generation.** We generate HDL based on the structure of the CaDG. Our hardware architecture is similar to how the brain is explained to work: Data are sent to the central neuron, which then distributes the data to the local neurons to be processed. In hardware, there are two types of nodes, i.e., the control and the compute nodes. On the one hand, there is a one-to-one correspondence between function calls and control nodes to transfer data into compute engines. On the other hand, we generate connected compute nodes in CaDGs to calculate the results based on the inputs from the upstreaming control nodes.

**Figure 9:**
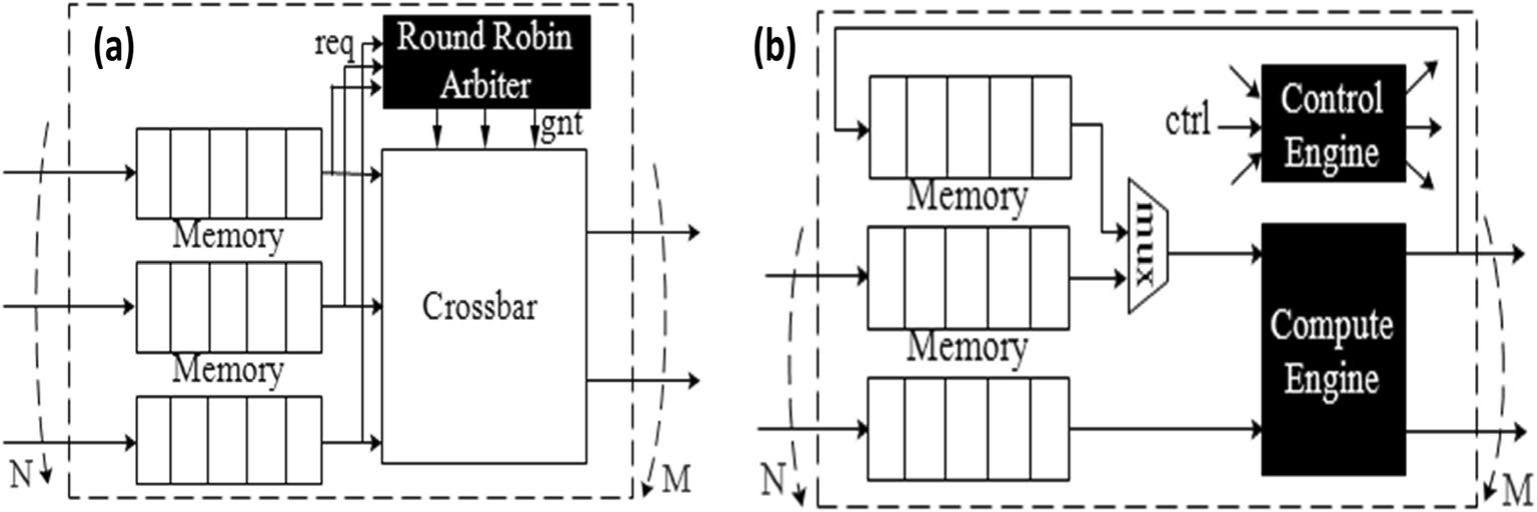
Hardware design template for (**a**) control nodes and (**b**) compute nodes.

*Control nodes.* The role of the control nodes is to buffer the incoming data, pipeline it and schedule it to the next available nodes. For instance, Fig. 9a illustrates a hardware design where buffers store message passing packets from previous nodes, a round-robin arbiter grants access to packets towards different ports (based on their requests), and a crossbar switch connects these buffers to the output ports.

*Compute nodes.* We generate HDL for different types of compute nodes as illustrated in Fig. 9b. A compute node requires the following components: buffers to buffer input data, one or more buffers connected with the compute engine to store temporary results from previous iterations, a *mux*/*demux* to arbitrate data to the compute engine, output ports, and a control engine. The control engine decides (1) when to push data into memories and pop data to the compute engine; (2) which output port data should be sent to; (3) whether data should be reused in loops. Depending on the number of iterations, compute engines are very resource intensive, meaning there are plenty of computations clustered together to form a large compute engine. Besides, due to loop unrolling, in hardware, repetitive patterns occur frequently, which means that it is highly parallel.

**Memory selection.** Memories act as abstract objects to ignore low-level hardware details. We propose the MemDSE framework for fast exploration of different memory architectures to optimize the performance and energy. The framework consists of memory architecture search including a metric for similarity between different architectures and acquisition function to guide the search for the optimum.

*Bayesian optimization for memory architecture search.* We define memory architecture search (MAS) as a technique for automating the design of memory architectures, an inseparable component in HPC. It includes the memory types, the memory sizes of different technologies, and the corresponding memory architecture. The goal is to find an optimal representation $x^*$ which maximizes a function *f* defined on the space of memory architectures ($x^*=\mathop {{\mathrm{arg\,max}}}\nolimits _{x}f(x)$). The function *f*, in the context of MAS, can be a combination of performance, power consumption, and bandwidth of a given representation *x*. We begin with a brief background on BO and further develop essential components for MAS.

*Bayesian optimization.* Machine learning techniques sometimes cannot efficiently solve the optimization formulation, e.g., $\max _x f(x)$ where the black-box function *f* does not have a closed-form expression, but can be evaluated at any arbitrary point *x* in the domain. This, in turn, generates a possibly noisy output *y* such that $E[y|f(x)]=f(x)$. Conversely, we can only obtain noisy observations *y* of the function *f*. This optimization problem can be efficiently addressed by BO^47,48^, which is a model-based technique to optimize a black-box function. In BO, prior beliefs *P*(*f*) over the possible objective functions are first defined and then sequentially refined from the likelihood $P(D_{1:t}|f)$ via Bayesian posterior updating $P(f|D_{1:t}) \propto P(f)P(D_{1:t}|f)$. The Bayesian posteriors denote our updated beliefs on the desired black-box objective function *f* we are optimizing given data $D_{1:t}$ collected from the model. Gaussian process (GP) priors are commonly used to model the objective function *f* in research^49–54^. A Gaussian process is a distribution over functions, completely specified by its prior mean function *m*(*x*), and covariance function $k(x,x')$.

10 $$\begin{aligned}{} & {} (f_1(x),..,f_t(x))^T \sim N(m(x), k(x,x')) \end{aligned}$$

11 $$\begin{aligned}{} & {} k(x,x')=e^{d^2(x,x')} \end{aligned}$$

For convenience, we assume that the prior mean is the zero function $m(x)=0$. In the context of MAS, the covariance function $k(x,x')$ represents a distance metric for similarity between different memory architectures *x* and $x'$.

With the help of the probabilistic model, we can define an acquisition function *u* which leverages the uncertainty in the posterior to guide exploration of the function *f* for the optimum. The acquisition function evaluates the utility of data $D_{1:t}$ for the next evaluation of *f*. Therefore, $x_{t+1}$ is selected by maximizing the acquisition function *u*, i.e., $x_{t+1} = \mathop {{\mathrm{arg\,max}}}\nolimits _x u(x|D_{1:t})$. The acquisition function, in the context of MAS, decides exploration and exploitation of the space of memory architectures with the help of program abstraction.

*Mathematical representation of memory architectures.* In order to quantify the similarity distance between different memory architectures, we first provide the definition of the mathematical formalism of memory architectures.

#### Definition 5

A memory architecture is represented as a weighted directed acyclic graph with node attributes to support the memory hierarchy $G \left( n^k_i, e^k_{ij}, w^k_{ij};n^k_i.attr|i,j\in {1,\ldots ,N}, k={0, \ldots , K}\right)$ where $n^k_i$ represents the *i*-th memory at the *k*-th depth associated with the corresponding attribute $n^k_i.attr$; the attribute $n^k_i.attr$ is a list of parameterized variables including the size, technology, whether it is volatile, density, cost, and power consumption listed in Table 1; $e^k_{ij}$ represents the interconnect between $n^k_i$ and $n^k_j$; *E* represents the memory topology which constitutes every possible $e^k_{ij}$; $w^k_{ij}$ represents a weight on an edge $e^k_{ij}$, which consists of bandwidth, speed, and latency; *N* and *K* represent the number of nodes and the largest path length, respectively.

*Distance metric for similarity between architectures.* In order to calculate the prior beliefs on memory architectures, we have to develop a distance metric to compare similarity between different architectures in the equation. Built on the mathematical representation of memory architectures, a relevant distance should capture different memory technologies, memory topology, and memory sizes. These factors partially determine the performance and energy consumption of programs running on top of the architecture, represented by the function *f*.

*Memory parameters.* In order to compare memory parameters such as technologies and sizes for different architectures, we encode each memory type into a real number and iterate every node at any depth to compare the difference as follows.

12 $$\begin{aligned} l_{mp}(x,x') = \sum _{k=1}^{K} \sum _{attr} \left| \mathscr {E}\left( n^k_i.attr_x \right) -\mathscr {E} \left( n^k_i.attr_{x'}\right) \right| \end{aligned}$$

where $n^k_i.attr_x$ represents the memory type at the *i*-th node of depth *k* and $\mathscr {E}$ is an encoder. For example, $l_{mp}(x,x')$ sums up the difference of memory technologies in any two architectures *x* and $x'$. This can help to identify the dissimilarity as the diameter of each graph is different from others. In addition, $l_{mp}(x,x')$ sums up the total difference of the memory sizes in terms of two architectures *x* and $x'$.

*Memory topology*: Memory topology is defined as the structure of interconnects between different memory technologies. It is either bus-based, mesh-based, or heterogeneous. The topology can be measured by the shortest path length of any two nodes in a graph. A path length of nodes *u* and *v* is the number of hops from one node to another in order to get from *u* to *v*. For example, the path length of DRAM and NVM is 0 due to no connections whereas (NVM, NVM, Flash) is a path from the node NVM to Flash of length 2. Therefore, we define another term as follows to take different memory topologies into consideration.

13 $$\begin{aligned} l_{mt}(x,x') = \left| \sum _{i\in x, j\in x}SPL(i,j)-\sum _{i'\in x', j'\in x'}SPL(i',j') \right| \end{aligned}$$

where SPL(i,j) calculates the shortest path length from nodes *i* to *j*. $l_{mt}(x,x')$ measures the difference between the sum of all shortest path in *x* and in $x'$, which can distinguish the topological difference between two architectures. The time complexity to calculate the shortest path length is $O(E+VlogV)$ where *E* is the number of edges and *V* is the number of nodes.

Putting it all together, the distance metric to compare similarity between different architectures for prior beliefs is as follows:

14 $$\begin{aligned} d(x,x') = -\sum _{g\in \{l_{mp},l_{mt}\}}g(x,x')log(g(x,x')) \end{aligned}$$

We apply the entropy to calculate the distance metric as it is a measure of the amount of information that is missing before reception. Once $d(x,x')$ is defined and calculated, our prior beliefs on a memory architecture *x* are available to calculate the posterior along with the likelihood function $P(D_{1:t}|f)$.

*Acquisition function.* So far we discuss how to construct the prior of memory architectures and measure the distance between any architectures. BO uses the prior and likelihood to construct the posterior for an acquisition function *u*(*t*) whose goal is to guide the search for the optimum. We maximize the acquisition function to select the next point $x_{t+1}$ at which to evaluate the function, i.e., $\mathop {{\mathrm{arg\,max}}}\nolimits u(x|D_{1:t})$.

Inspired by maximizing the probability of improvement^47^ over the incumbent $f(x+)$, where $x+ = \mathop {{\mathrm{arg\,max}}}\nolimits _{x_i\in x_{1:t}} f(x_i)$, we propose the following probability over the possible memory architecture *x*:

15 $$\begin{aligned} \begin{aligned} {PI'(x)}&= P(f(x)\ge f(x+))\\&= \Phi \left( \frac{\mu (x)-f(x+)-\lambda (st|x+)}{\sigma (x)}\right) \end{aligned} \end{aligned}$$

where $\mu (x)$ and $\sigma (x)$ represent the GP mean and standard deviation of prediction at a point *x*, respectively; *st* is a vector of statistics collected in each CaDG; $\lambda ()$ is a term we introduce to overcome the drawback of the probability of improvement. Without the $\lambda (st|x+)$ term, the Eq. (15) is pure exploitation, meaning that points that have a high probability of being greater than $f(x+)$ will be drawn. However, with the help of program statistics, we can design $\lambda (st|x+)$ as follows to explore more regions of appropriate memory types.

$$\begin{aligned} \lambda (st|x+) = {\left\{ \begin{array}{ll} \frac{\partial f(x)}{\partial x.t}\vert _{x+} &{} \text {if st.lpl}\gg \text {st.apl or st.spl}\ll \text {st.apl}\\ \frac{\partial f(x)}{\partial x.3d}\vert _{x+} &{} \text {elif st.read+st.write>st.cmpt}\\ \frac{\partial f(x)}{\partial x.cache}\vert _{x+} &{} \text {elif st.edges>N}\\ 10^3-0.01t &{} \text {otherwise} \end{array}\right. } \end{aligned}$$

where *x*.*t*, *x*.3*d*, and *x*.*cache* represent parameters in the topology, 3D-stacked memory, and cache to the main memory, respectively; *st*.*lpl*, *st*.*apl*, *st*.*spl*, *st*.*read*, *st*.*write*, *st*.*cmpt*, *st*.*edges*, and *N* represent the longest/average/shortest path lengths (abbreviated as lpl, apl, and spl), the number of read/write/compute operations, the number of edges, and the number of nodes, respectively. Since the function *f* does not have an analytical form, we approximate the derivative as $\frac{\partial f(x)}{\partial x}\vert _{x+}\approx \Delta f(x+1)-\Delta f(x)|_{x+}$.

If lpl is much greater than apl or spl is much smaller than apl, we believe that the search should explore regions of different topologies as indicated by $\partial x.t$. If the number of memory operations is greater than the number of computations, the guided search may visit more regions of 3D-stacked memory to provide high bandwidth. If the number of edges is greater than the number of nodes, data communication dominates and the search should traverse regions of cache to the main memory to provide the fast accesses. Otherwise, we maintain a schedule for $\lambda (st|x+)$, so that it starts high early in the optimization, to drive exploration of any possible regions, and decreases to zero afterwards in order to keep exploitation.

## Data Availability

The datasets used and/or analysed during the current study are available from the corresponding author on reasonable request.
